# Supplementary material for: Alt-RPL36 downregulates the PI3K-AKT-mTOR signaling pathway by interacting with TMEM24
Source: Nat Commun. 2021 Jan 21;12:508. doi: 10.1038/s41467-020-20841-6 (PMC7820019; doi:10.1038/s41467-020-20841-6)
Supplement: Supplementary file 1 — Supplementary Information [file 41467_2020_20841_MOESM1_ESM.pdf]

Supplementary Information

Alt-RPL36 downregulates the PI3K-AKT-mTOR signaling pathway by interacting with TMEM24

Xiongwen Cao[1,2,5], Alexandra Khitun[1,2,5], Yang Luo[1,2], Zhenkun Na[1,2], Thitima Phoodokmai[3], Khomkrit Sappakhaw[3], Elizabeth Olatunji[2], Chayasith Uttamapinant[3], Sarah A. Slavoff[1,2,4*]

[1]Department of Chemistry, Yale University, New Haven, Connecticut 06520, United States

[2]Chemical Biology Institute, Yale University, West Haven, Connecticut 06516, United States

[3]School of Biomolecular Science and Engineering, Vidyasirimedhi Institute of Science and Technology (VISTEC), Rayong, Thailand

[4]Department of Molecular Biophysics and Biochemistry, Yale University, New Haven, Connecticut 06529, United States

[5]These authors contributed equally

*Correspondence: sarah.slavoff@yale.edu

a

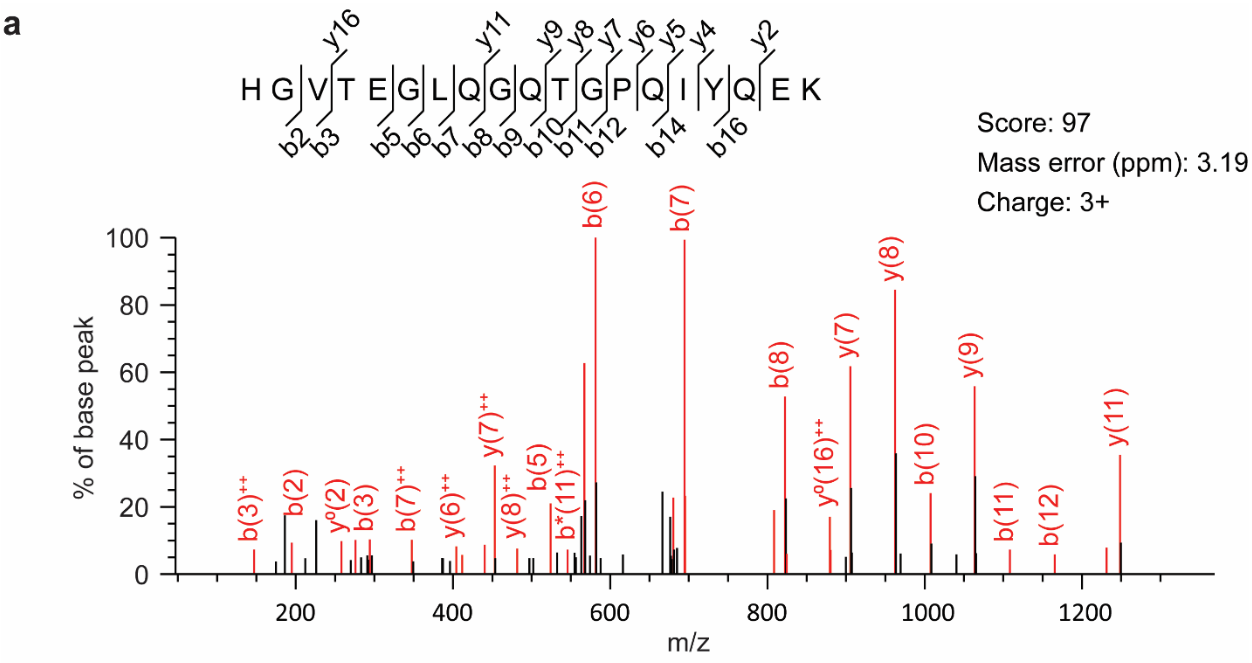

b

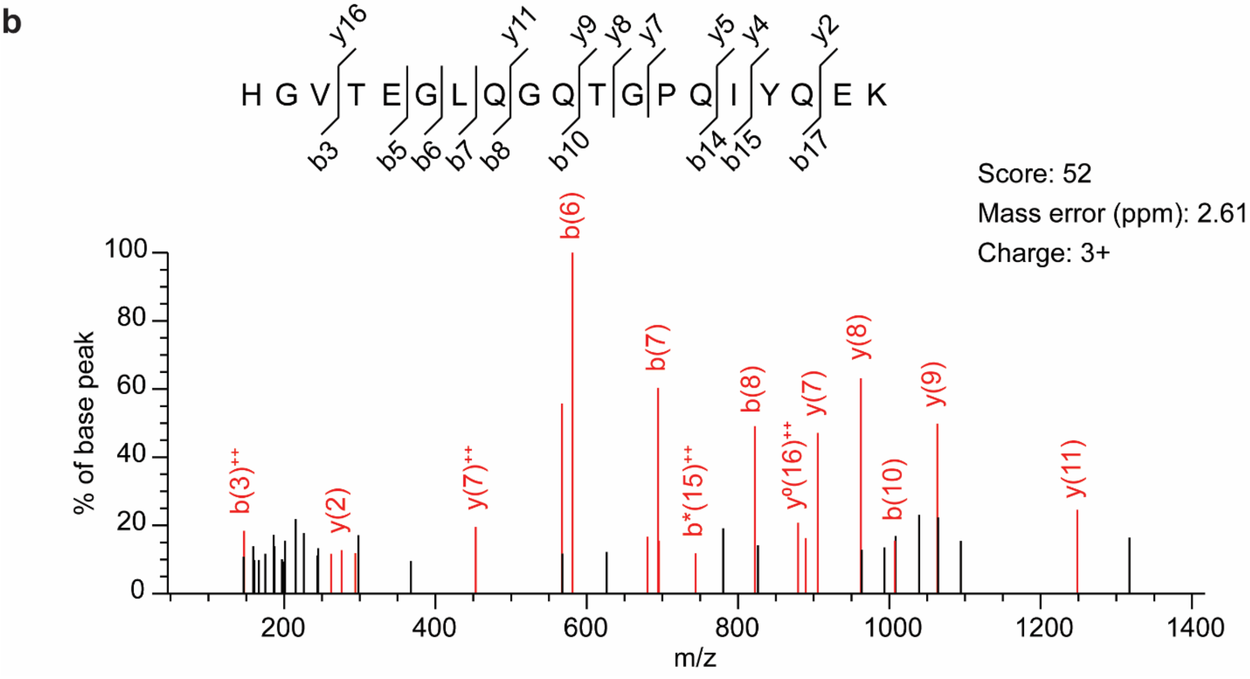

Supplementary Fig. 1 Alt-RPL36 is expressed in HT1080 and MOLT4 cells. MS/MS spectra of a unique alt-RPL36 tryptic peptide detected via peptidomics in HT1080 cells (**a**) and MOLT4 cells (**b**).

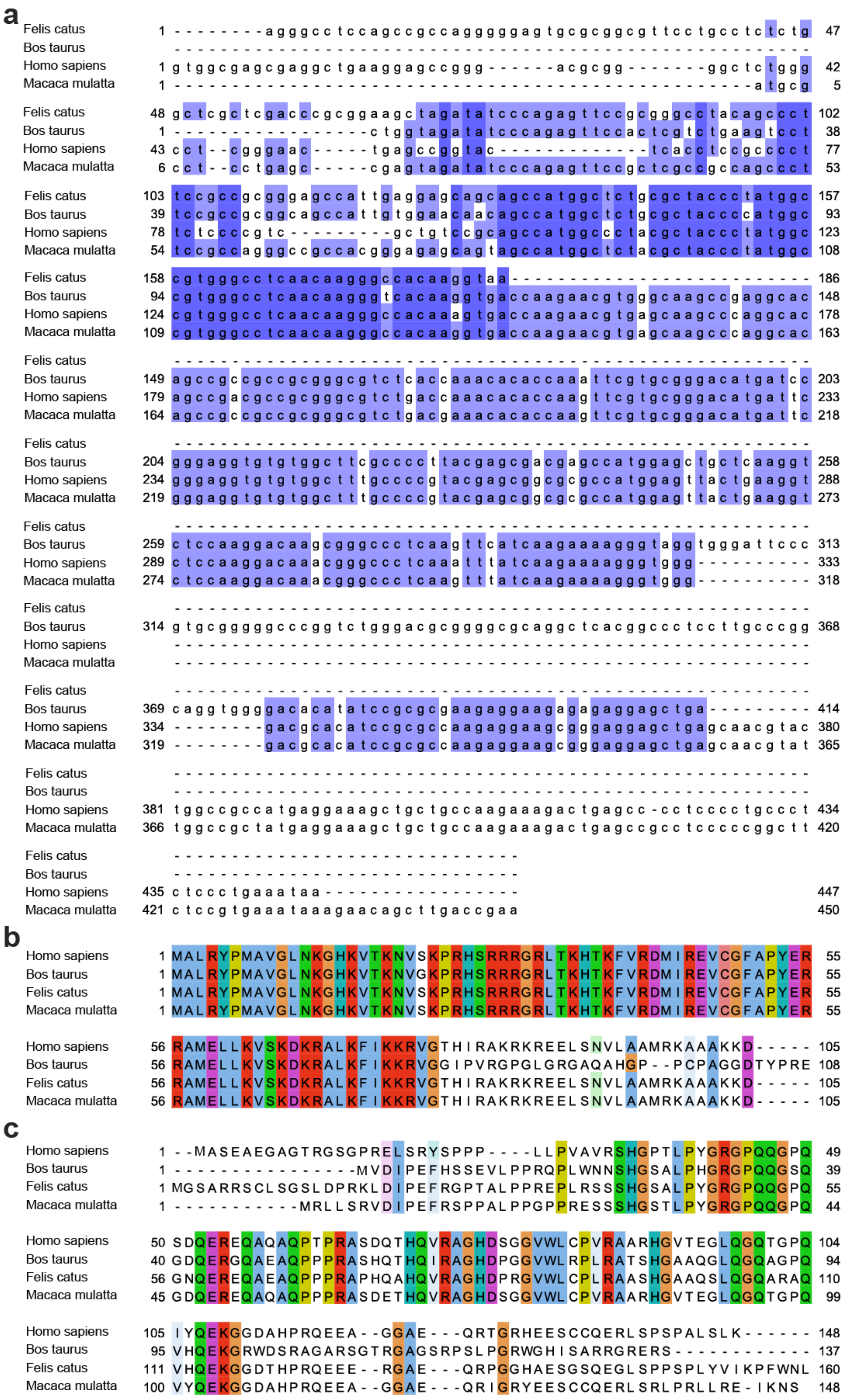
a
Felis catus 1 --------agggcctccagccgccaggggggagtgcgcggcgttcctgcctctctg 47
Bos taurus --
Homo sapiens 1 gtggcgagcgaggctgaaggagccggg------acgcgg-------ggctctggg 42
Macaca mulatta 1 ---atgcg 5
Felis catus 48 gctcgctcgaccgcggaagctagatatcccagagttccgcgggcctacagccct 102
Bos taurus 1 ----------------ctggtagatatcccagagttccactcgtctgaagtcct 38
Homo sapiens 43 cct--cgggaac-----tgagccggtac-------------tcacctccgccct 77
Macaca mulatta 6 cct--cctgagc-----cgagtagatatcccagagttccgctcgccgccagccct 53
Felis catus 103 tcgccgcgggagccattgaggagcagcagccatggctctgcgctaccctatggc 157
Bos taurus 39 tcgccgcggcagccattgtggaacaacagccatggctctgcgctaccccatggc 93
Homo sapiens 78 tctcccgtc---------gctgtccgcagccatggccctacgctaccctatggc 123
Macaca mulatta 54 tcgccaggccgccacgggagagcagtagccatggctctacgctaccctatggc 108
Felis catus 158 cgtggcctcaacaagggccacaaggtaa--------------------------- 186
Bos taurus 94 cgtggcctcaacaagggtcacaaggtgaccaagaacgtgggcaagccgaggcac 148
Homo sapiens 124 cgtggcctcaacaagggccacaaagtgaccaagaacgtgagcaagcccaggcac 178
Macaca mulatta 109 cgtggcctcaacaagggccacaaggtgaccaagaacgtgagcaagcccaggcac 163
Felis catus ---
Bos taurus 149 agccgccgccgcgggcgtctcaccaaacacaccaaattcgtgcgggacatgatcc 203
Homo sapiens 179 agccgacgccgcgggcgtctgaccaaacacaccaagttcgtgcgggacatgattc 233
Macaca mulatta 164 agccgccgccgcgggcgtctgacgaaacacaccaagttcgtgcgggacatgattc 218
Felis catus ---
Bos taurus 204 gggaggtgtgtggcttcgcccctacgagcgacgagccatggagctgctcaaggt 258
Homo sapiens 234 gggaggtgtgtggcttgccccgtacgagcggcgcgccatggagttactgaaggt 288
Macaca mulatta 219 gggaggtgtgtggcttgccccgtacgagcggcgcgccatggagttactgaaggt 273
Felis catus ---
Bos taurus 259 ctccaaggacaagcggccctcaagttcatcaagaaagggtaggtgggattccc 313
Homo sapiens 289 ctccaaggacaaacggccctcaaatttatcaagaaagggtggg---------- 333
Macaca mulatta 274 ctccaaggacaaacggccctcaagtttatcaagaaagggtggg---------- 318
Felis catus ---
Bos taurus 314 gtgcgggggcccggtctgggacgcggggcgcaggctcacggccctccttgcccgg 368
Homo sapiens ---
Macaca mulatta ---
Felis catus ---
Bos taurus 369 caggtggggacacatatccgcgcgaagaggaagagaggaggagctga--------- 414
Homo sapiens 334 --------gacgcacatccgcgccaagaggaagcgggaggagctgagcaacgtac 380
Macaca mulatta 319 --------gacgcacatccgcgccaagaggaagcgggaggagctgagcaacgtat 365
Felis catus ---
Bos taurus ---
Homo sapiens 381 tggccgccatgaggaaagctgctgccaagaaagactgagcc-cctcccctgccct 434
Macaca mulatta 366 tggccgctatgaggaaagctgctgccaagaaagactgagccgcctcccccggctt 420
Felis catus ------------------------------
Bos taurus ------------------------------
Homo sapiens 435 ctccctgaaataa----------------- 447
Macaca mulatta 421 ctccgtgaaataaagaacagcttgaccgaa 450
b
Homo sapiens 1 MALRYPMAVGLNKGHKVTKNVSKPRHSRRRGRLTKHTKFVRDMIREVCGFAPYER 55
Bos taurus 1 MALRYPMAVGLNKGHKVTKNVGKPRHSRRRGRLTKHTKFVRDMIREVCGFAPYER 55
Felis catus 1 MALRYPMAVGLNKGHKVTKNVSKPRHSRRRGRLTKHTKFVRDMIREVCGFAPYER 55
Macaca mulatta 1 MALRYPMAVGLNKGHKVTKNVSKPRHSRRRGRLTKHTKFVRDMIREVCGFAPYER 55
Homo sapiens 56 RAMELLKVSKDKRALKFIKKRVGTHIRAKRKREELSNVLAAMRKAAAKKD----- 105
Bos taurus 56 RAMELLKVSKDKRALKFIKKRVGGIPVRGPGLGRGAQAHGP--PCPAGGDTYPRE 108
Felis catus 56 RAMELLKVSKDKRALKFIKKRVGTHIRAKRKREELSNVLAAMRKAAAKKD----- 105
Macaca mulatta 56 RAMELLKVSKDKRALKFIKKRVGTHIRAKRKREELSNVLAAMRKAAAKKD----- 105
c
Homo sapiens 1 --MASEAEGAGTRGSGPRELSRYSPPP----LLPVAVRSHGPTLPYGRGPQQGPQ 49
Bos taurus 1 ----------------MVDIPEFHSSEVLPPRQPLWNNSHGSALPHGRGPQQGSQ 39
Felis catus 1 MGSARRSCLSGSLDPRKLDIPEFRGPTALPPREPLRSSSHGSALPYGRGPQQGPQ 55
Macaca mulatta 1 -----------MRLLSRVDIPEFRSPPALPPGPPRESSSHGSTLPYGRGPQQGPQ 44
Homo sapiens 50 SDQEREQAQAQPTPRASDQTHQVRAGHDSGGVWLCPVRAARHGVTEGLQGQTGPQ 104
Bos taurus 40 GDQERGQAEAQPPPRASHQTHQIRAGHDPGGVWLRPLRATSHGAAQGLQGQAGPQ 94
Felis catus 56 GNQEREQAEAQPPPRAPHQAHQVRAGHDPRGVWLCPLRAASHGAAQSLQGQARAQ 110
Macaca mulatta 45 GDQEREQAQAQPPPRASDETHQVRAGHDSGGVWLCPVRAARHGVTEGLQGQTGPQ 99
Homo sapiens 105 IYQEKGGDAHPRQEEA--GGAE---QRTGRHEESCCQERLSPSPALSLK------ 148
Bos taurus 95 VHQEKGRWDSRAGARSGTRGAGSRPSLPGRWGHISARRGRERS------------ 137
Felis catus 111 VHQEKGGDTHPRQEEE--RGAE---QRPGGHAESGSQEGLSPPSPLYVIKPFWNL 160
Macaca mulatta 100 VYQEKGGDAHPRQEEA--GGAE---QRIGRYEESCCQERLSRLPRLLRE-IKNS 148

Supplementary Fig. 2 Alt-RPL36 is predicted to be conserved in some mammals. a mRNA sequence conservation of RPL36 transcript isoforms in *Bos taurus* (XR_003035408.1), *Felis catus* (XM_011287701.3), and *Macaca mulatta* (XM_015122577.2) at the nucleotide level. Conserved nucleotides are highlighted in blue. Dark blue indicates identity, while light blue indicates conservation in at least 3 of 4 species. Species with cognate or near-cognate start codons in strong Kozak consensus sequence in the alt-RPL36 reading frame were chosen for analysis. **b-c** ClustalW2 alignment of RPL36 (**b**) and alt-RPL36 (**c**) protein sequences translated from transcripts shown in (**a**), residues are highlighted to indicate identity. ClustalW2 alignments were visualized using JalView.

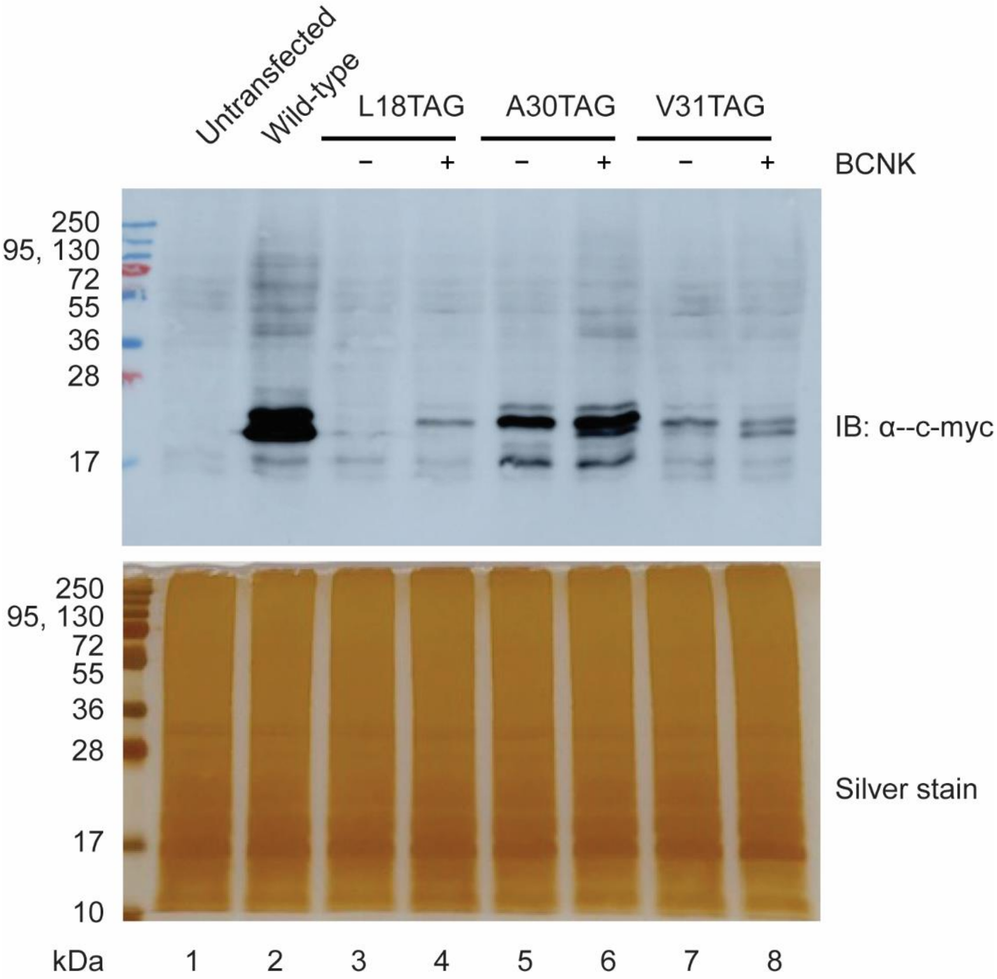

Supplementary Fig. 3 Expression of alt-RPL36 variants via genetic code expansion. HEK 293 cells were transfected with transgenes encoding *M. mazei* pyrrolysyl-tRNA synthetase bearing Y306A/Y384F mutations (PylRS-AF), amber-suppressing Pyl tRNA (PylTCUA), and variants of amber codon (TAG)-bearing, c-myc-tagged alt-RPL36. Cells were incubated in the presence or absence of 60 μM bicyclononyne-lysine (BCNK) for 45 hours at 37℃, lysed, and analyzed by Western blotting with anti-c-myc antibody (upper), silver stain served as a loading control (lower). Controls with untransfected cells (lane 1) and cells expressing wild-type alt-RPL36 (lane 2) are shown.

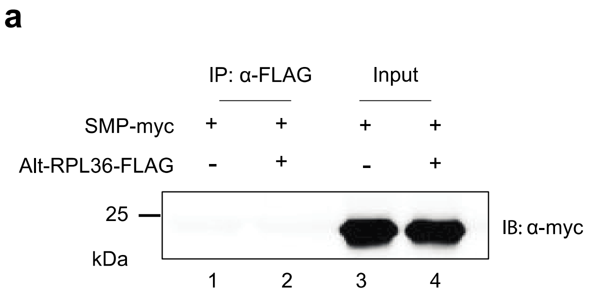

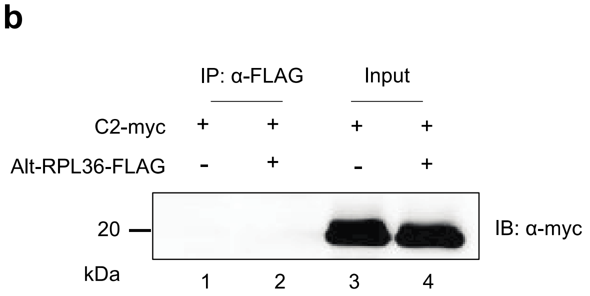

Supplementary Fig. 4 Both the SMP and the C2 domain are required for the interaction of TMEM24 with alt-RPL36. a and **b** Control HEK 293T cells (lane 1) or HEK 293T cells stably expressing alt-RPL36-FLAG-HA (lane 2) were transfected with the indicated TMEM24 truncation mutants, and immunoprecipitation (IP) was performed with anti-FLAG antibody, followed by immunoblotting (IB) with anti-myc antibody. Cell lysates (1%) before IP (input, lanes 3 and 4) were used as the loading controls. Data are representative of three biological replicates.

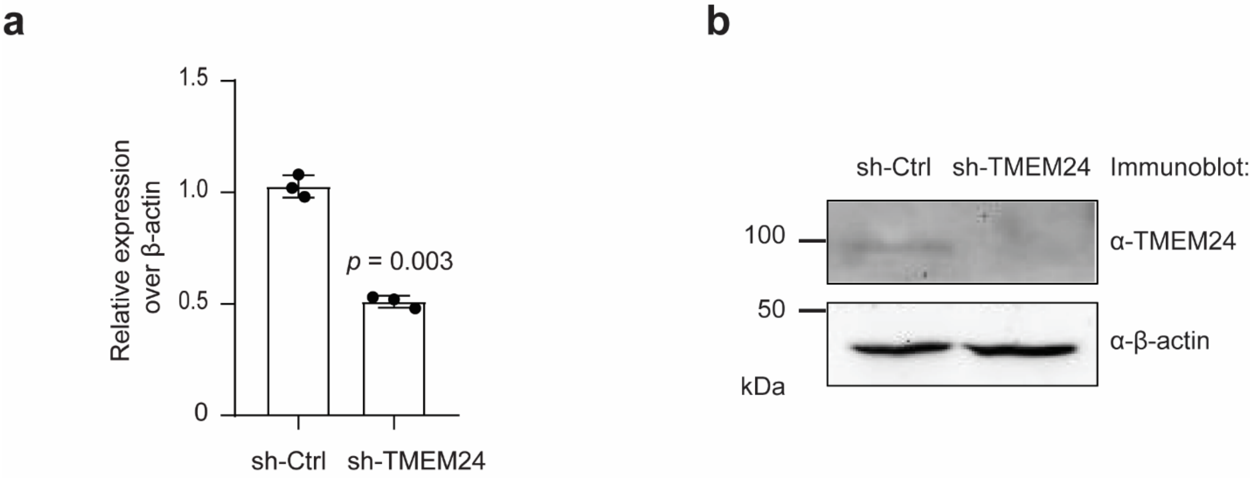

Supplementary Fig. 5 Silencing TMEM24 in alt-RPL36-FLAG knock-in (KI) cells. a Quantitative RT-PCR of alt-RPL36 KI cells stably expressing control shRNA (sh-Ctrl) or TMEM24 shRNA (sh-TMEM24) with primer specific to TMEM24 (error bars, standard error of the mean (s.e.m.), N = 3 biologically independent samples, two-tailed t-test. Source data are provided as a Source Data file. **b** Western blot analysis of alt-RPL36 KI cells stably expressing control shRNA (sh-Ctrl) or TMEM24 shRNA (sh-TMEM24) with the indicated antibodies. Data are representative of two biological replicates.

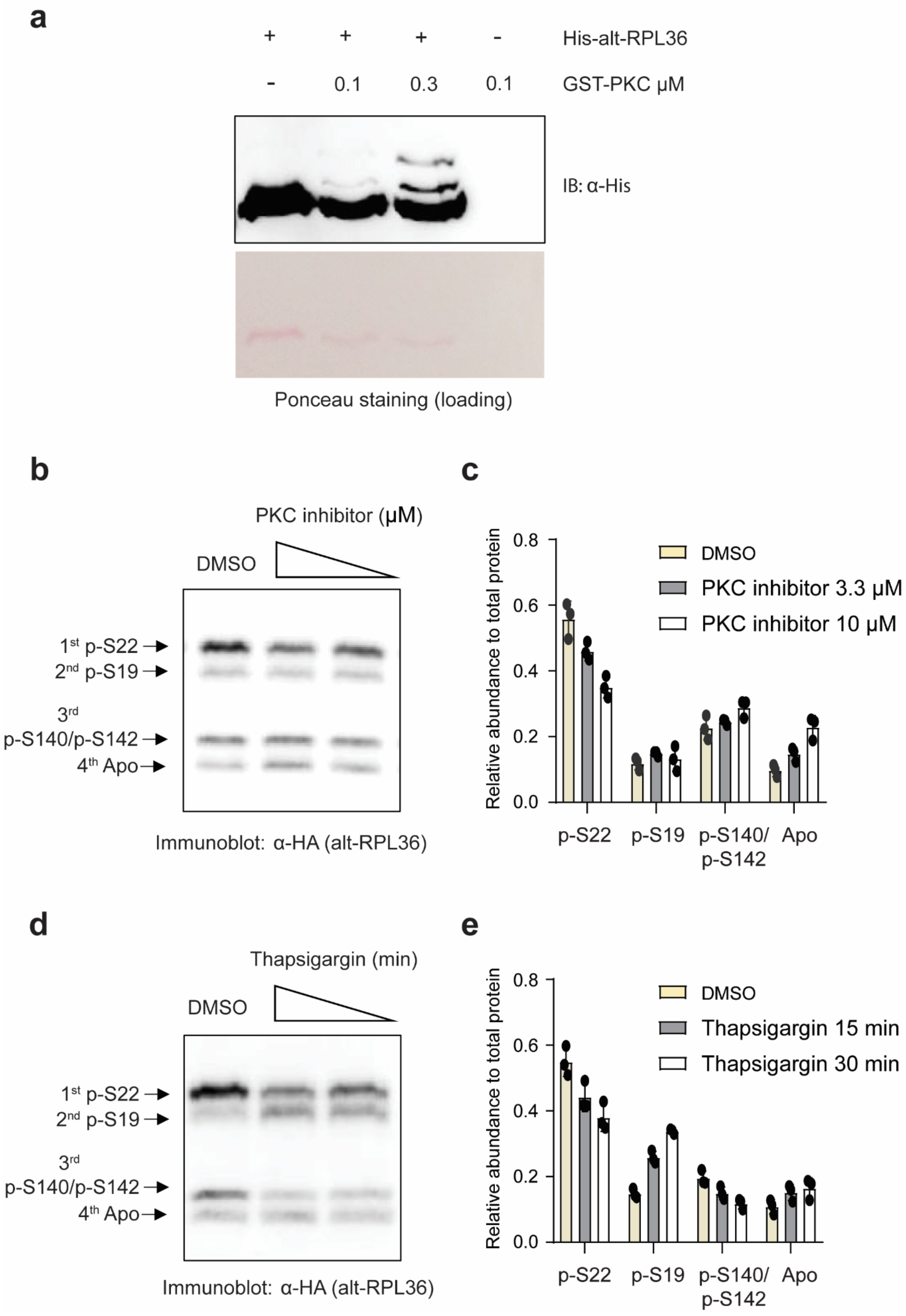
a
His-alt-RPL36
GST-PKC µM
+ + + -
- 0.1 0.3 0.1
IB: α-His
Ponceau staining (loading)
b
PKC inhibitor (µM)
DMSO
1st p-S22
2nd p-S19
3rd p-S140/p-S142
4th Apo
Immunoblot: α-HA (alt-RPL36)
c
Relative abundance to total protein
0.0
0.2
0.4
0.6
0.8
DMSO
PKC inhibitor 3.3 µM
PKC inhibitor 10 µM
p-S22
p-S19
p-S140/p-S142
Apo
d
Thapsigargin (min)
DMSO
1st p-S22
2nd p-S19
3rd p-S140/p-S142
4th Apo
Immunoblot: α-HA (alt-RPL36)
e
Relative abundance to total protein
0.0
0.2
0.4
0.6
0.8
DMSO
Thapsigargin 15 min
Thapsigargin 30 min
p-S22
p-S19
p-S140/p-S142
Apo

Supplementary Fig. 6 Phosphorylation of alt-RPL36 is regulated by protein kinase C (PKC) and cytosolic calcium level. a Purified alt-RPL36 was allowed to react with increasing amounts of PKC, and the reaction was analyzed by Phos-tag Western blotting with anti-His tag antibody. Ponceau staining served as a loading control. **b** HEK 293T cells stably expressing alt-RPL36-FLAG-HA were treated with increasing amounts of PKC inhibitor or vehicle for 8 hr, followed with Phos-tag Western blotting. **c** Quantitative analysis of the Western blot signal of the bands in (**b**). Data represent mean values ± standard error of the mean (s.e.m.) of three biological replicates. Source data are provided as a Source Data file. **d** HEK 293T cells stably expressing alt-RPL36-FLAG-HA were treated with 1 μM thapsigargin for different times or vehicle, followed with Phos-tag Western blotting. **e** Quantitative analysis of the Western blot signal of the bands in (**d**). Data represent mean values ± standard error of the mean (s.e.m.) of three biological replicates. Source data are provided as a Source Data file.

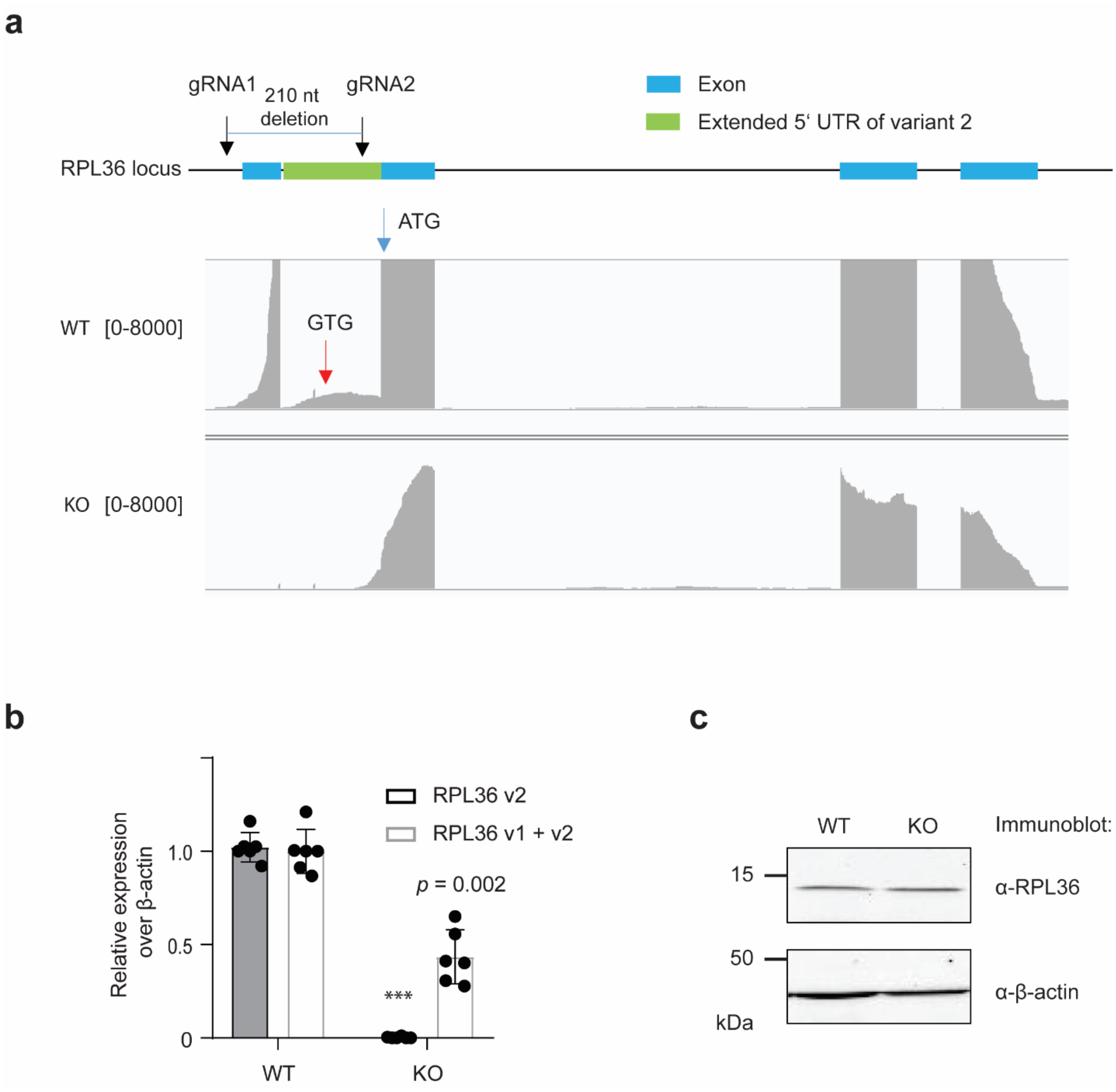

Supplementary Fig. 7 Validation of the alt-RPL36 knock-out HEK 293T cell line. a Top, schematic representation of the human *RPL36* genomic locus. The first (blue) exon is specific to transcript variant 1; the second (green) exon is specific to transcript variant 2 and encodes the alt-RPL36 start codon; downstream blue exons are shared by both transcript variants and encode the annotated RPL36 coding sequence. Bottom, RNA-seq reads mapping to the *RPL36* genomic locus in wild-type (WT) and alt-RPL36 knock-out (KO) cells. The position of the GTG start codon of alt-RPL36 is indicated with a red arrow, and the position of the ATG start codon of RPL36 is indicated with a blue arrow. The y-axis scale for RNA is reads per kilobase per million reads (y-axis scale, 0-8000 RPKM). **b** Quantitative RT-PCR with primers specific to RPL36 variant 2 (RPL36 v2) or primers that detect both variants (RPL36 v1 and v2) (error bars,

standard error of the mean (s.e.m.), $N = 6$ biologically independent samples, *** $p < 0.001$ (two-tailed *t*-test)). Source data are provided as a Source Data file. **c** Western blot analysis of the wild-type and alt-RPL36 knock-out HEK 293T cells with the indicated antibodies. Data are representative of three biological replicates.

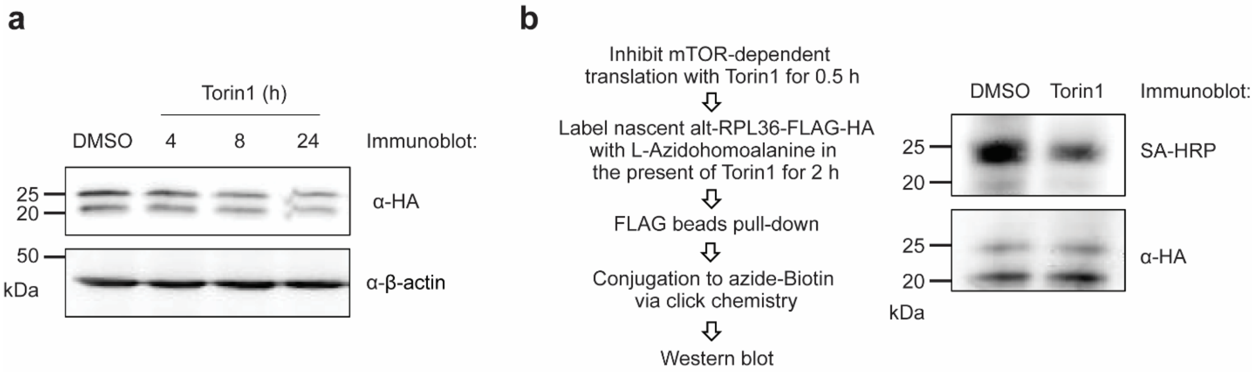

Supplementary Fig. 8 mTOR inhibition downregulates the translation of alt-RPL36. a HEK 293T cells stably expressing alt-RPL36-FLAG-HA were treated with 0.5 μM mTOR inhibitor Torin1 for different times or vehicle, followed with Western blot. Data are representative of three biological replicates. **b** Left: experimental scheme for newly translated alt-RPL36-FLAG-HA capture. Right: anti-HA reflects the total protein level of alt-RPL36-FLAG-HA, and streptavidin-HRP (SA-HRP) reflects newly synthesized alt-RPL36-FLAG-HA. Data are representative of two biological replicates.

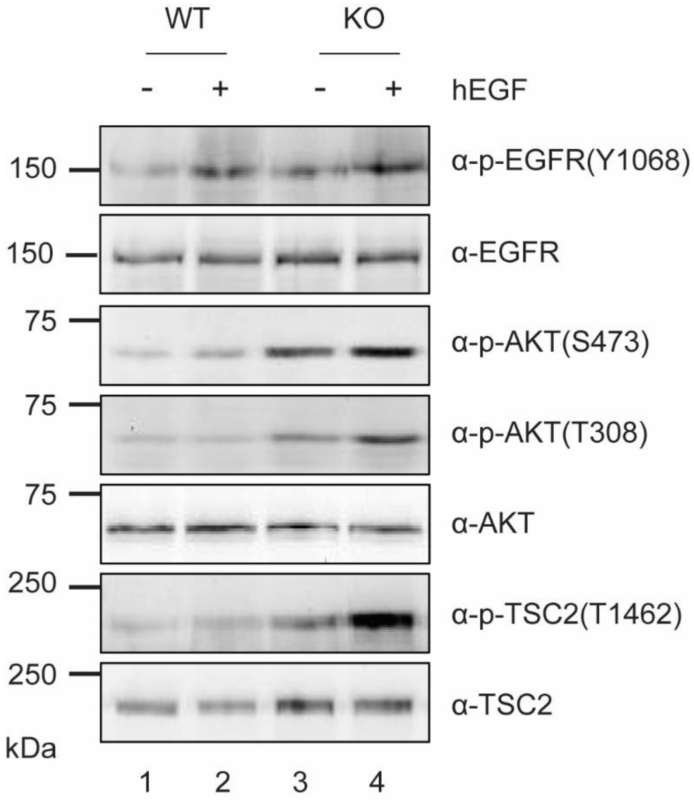

Supplementary Fig. 9 Alt-RPL36 knock-out cells are more sensitive to EGF stimulation than wild-type cells. Wild-type (WT) or alt-RPL36 knock-out (KO) HEK 293T cells were treated with FBS-free media for 6 h, followed by 100 ng/mL hEGF stimulation for 15 min or control (no stimulation). Cells were collected, and Western blot performed with the antibodies indicated on the right. All Western blots are representative of two biological replicates.

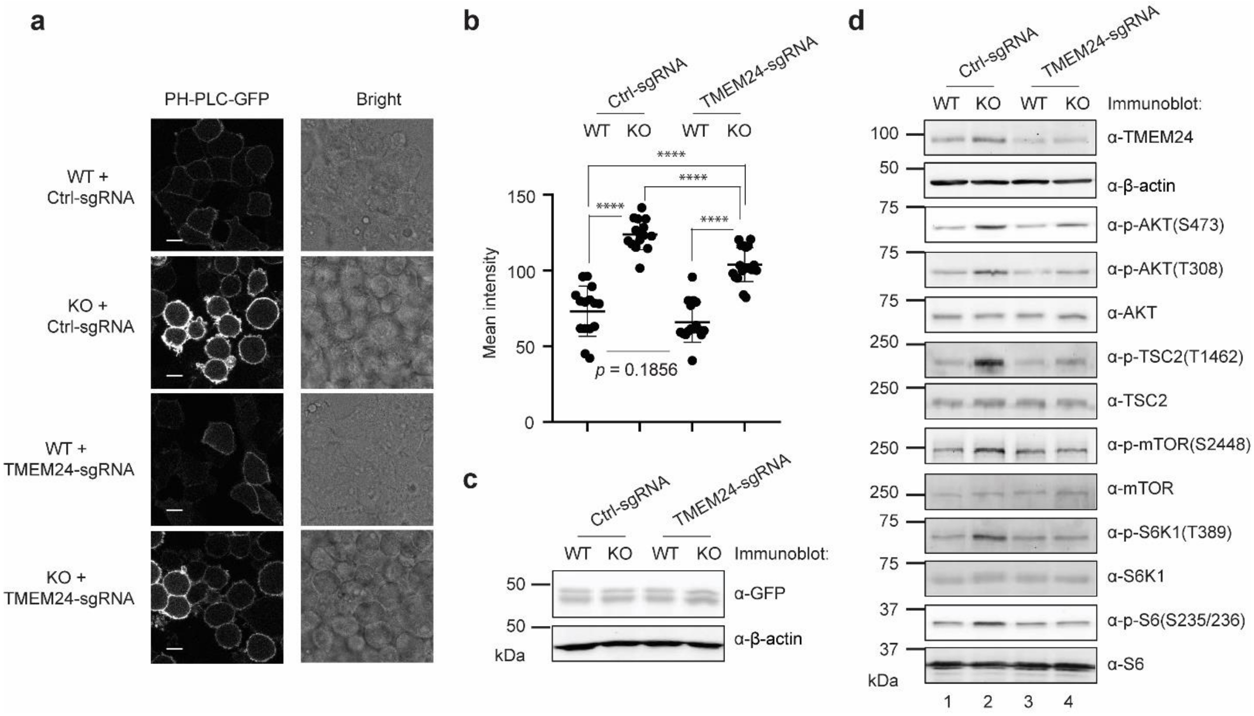

Supplementary Fig. 10 Regulation of PI3K-AKT-mTOR pathway by alt-RPL36 requires its interaction with TMEM24. a PH-PLC-GFP reporter was stably introduced into the following cell lines: HEK 293T cells stably expressing Cas9 and control sgRNA (WT + Ctrl-sgRNA), alt-RPL36 KO cells stably expressing Cas9 and control sgRNA (KO + Ctrl-sgRNA), HEK 293T stably expressing Cas9 and TMEM24 sgRNA (WT + TMEM24-sgRNA), alt-RPL36 KO stably expressing Cas9 and TMEM24 sgRNA (KO + TMEM24-sgRNA). Confocal imaging was performed with live cells. Scale bar, 10 µm. **b** Quantitation of the PH-PLC-GFP signals in the four cell lines described above. At least 15 fields of view were analyzed, totaling > 300 cells for each measurement. Significance was evaluated with two-tailed *t*-test. **** $p < 0.0001$. Data are representative of three biological replicates. Source data are provided as a Source Data file. **c** Western blot of the four cell lines with antibodies indicated on the right for comparison of PH-PLC-GFP expression. Data are representative of three biological replicates. **d** Western blot analysis of the four cell lines described above with antibodies indicated on the right. Data are representative of three biological replicates.

a

Mutation data obtained from COSMIC			
CDS mutation	RPL36 AA mutation	alt-RPL36 AA mutation	Primary Tissue
49G>A	V17M	S50N	Skin
194A>C	K65T	Q98H	Large intestine
212A>C	K71T	Q104H	Urinary tract
243C>G	I81M	P115A	Breast
254G>A	R85K	Synonymous	Large intestine
284C>T	A95V	Synonymous	Stomach
117C>T	Synonymous	R73C	Upper aerodigestive tract
117C>T	Synonymous	R73C	Urinary tract
178T>C	Synonymous	V93A	Liver
252G>A	Synonymous	E118K	Cervix

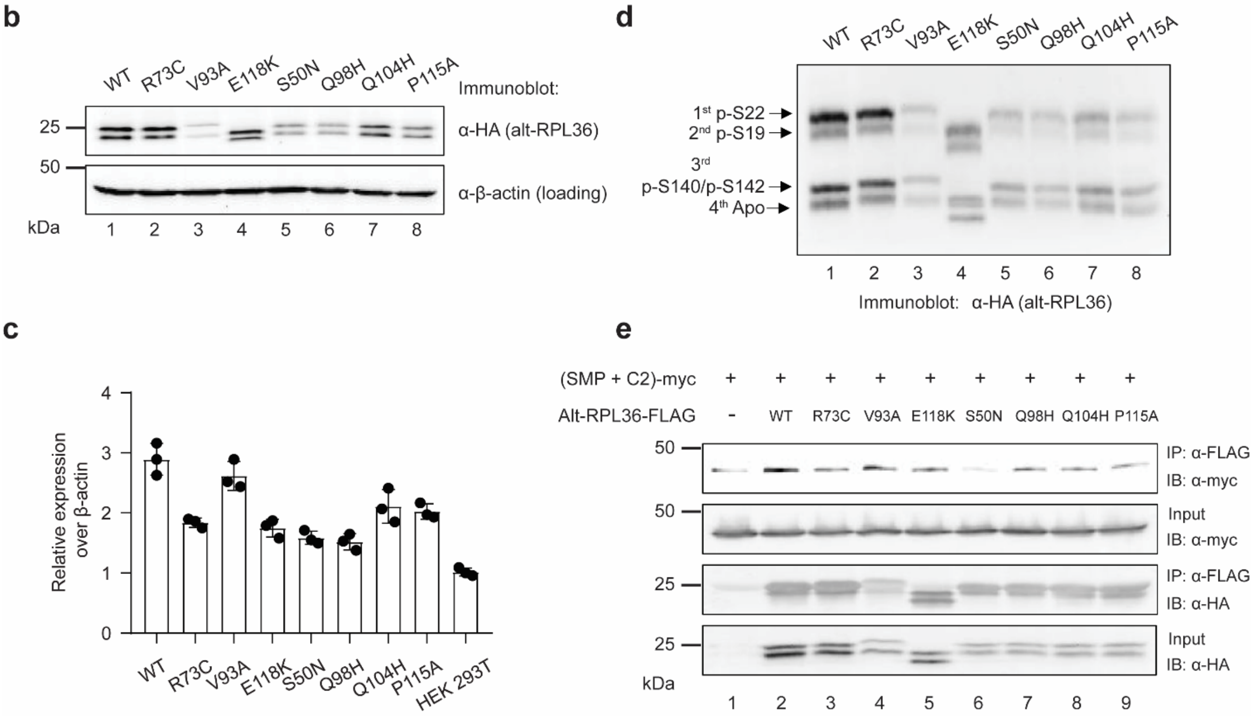

Supplementary Fig. 11 Cancer-related mutations affect alt-RPL36 protein properties and interaction. a Cancer-associated non-synonymous mutations in *RPL36* and/or *alt-RPL36* from the COSMIC database. AA, amino acid. COSMIC DNA numbering is relative to the *RPL36* start codon (A_1TG). **b** SDS-PAGE Western blot of HEK 293T cells stably expressing wild-type (WT) alt-RPL36 or point mutants indicated at the top with antibodies indicated on the right. **c** Quantitative RT-PCR of control HEK 293T cells (HEK 293T) or the stable cell lines indicated at the bottom using primers designed against *RPL36* transcript variant 2 that are specific to the alt-RPL36 coding sequence (error bars, standard error of the mean (s.e.m.), *N* = 3 biologically independent samples. Source data are provided as a Source Data file. **d** Wild-type alt-RPL36-FLAG-HA or point mutants indicated at the top were immunopurified from HEK 293T cells, resolved with Phos-tag SDS-PAGE, and detected with anti-HA via Western blotting. **e** Control HEK 293T cells (lane 1), HEK 293T cells stably expressing wild-type alt-RPL36-FLAG-HA (lane

2), and HEK 293T cells stably expressing point mutants indicated at the top (lane 3 to 9) were transfected with the TMEM24 SMP + C2 domain construct, followed by IP with anti-FLAG antibody and immunoblotting with antibodies indicated on the right. Cell lysates (1%) before IP (input) were used as loading controls. All Western blots are representative of two biological replicates.

a

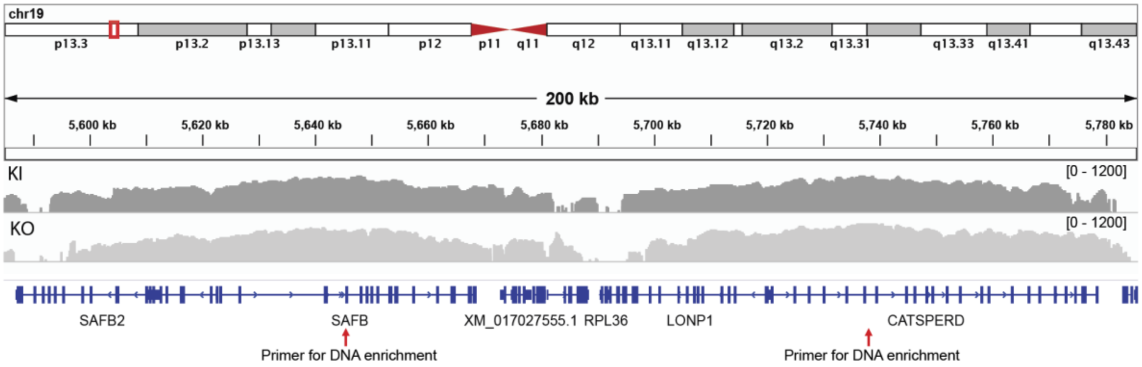

b

Chromosome	Position	Cell Line	Type of variant	Supporting Reads	Gene	Intron/Exon
chr19	5708507	KO	Insertion	125	LONP1	Intron
chr19	5740515-5742010	KO	Inversion	20	CATSPERD	Intron
chr19	5741045-5741079	KO	Deletion	376	CATSPERD	Intron
chr19	5761620	KO	Insertion	15	CATSPERD	Intron
chr19	5708507	KI	Insertion	81	LONP1	Intron
chr19	5741045-5741079	KI	Deletion	235	CATSPERD	Intron
chr19	5761620	KI	Insertion	25	CATSPERD	Intron

Supplementary Fig. 12 No large deletions or insertions were observed in the 100 kb upstream or 100 kb downstream of RPL36 in the alt-RPL36 knock-out and knock-in cell lines. Shown are the aligned Xdrop sequencing results targeting 100 kb upstream and 100 kb downstream of RPL36 (**a**), visualized via IGV. The primer sites selected for targeted DNA enrichment are indicated with a red arrow. Four structural variants were detected in the targeted region (**b**), three of which are shared by the knock-out and knock-in cell lines, and one inversion specific to the knock-out cell line localized to an intronic sequence within the CATSPERD gene. No cell-line specific insertions or deletions were detected. We did not observe the inserted sequence in the knock-in cells, possibly because of low coverage of the RPL36 gene.

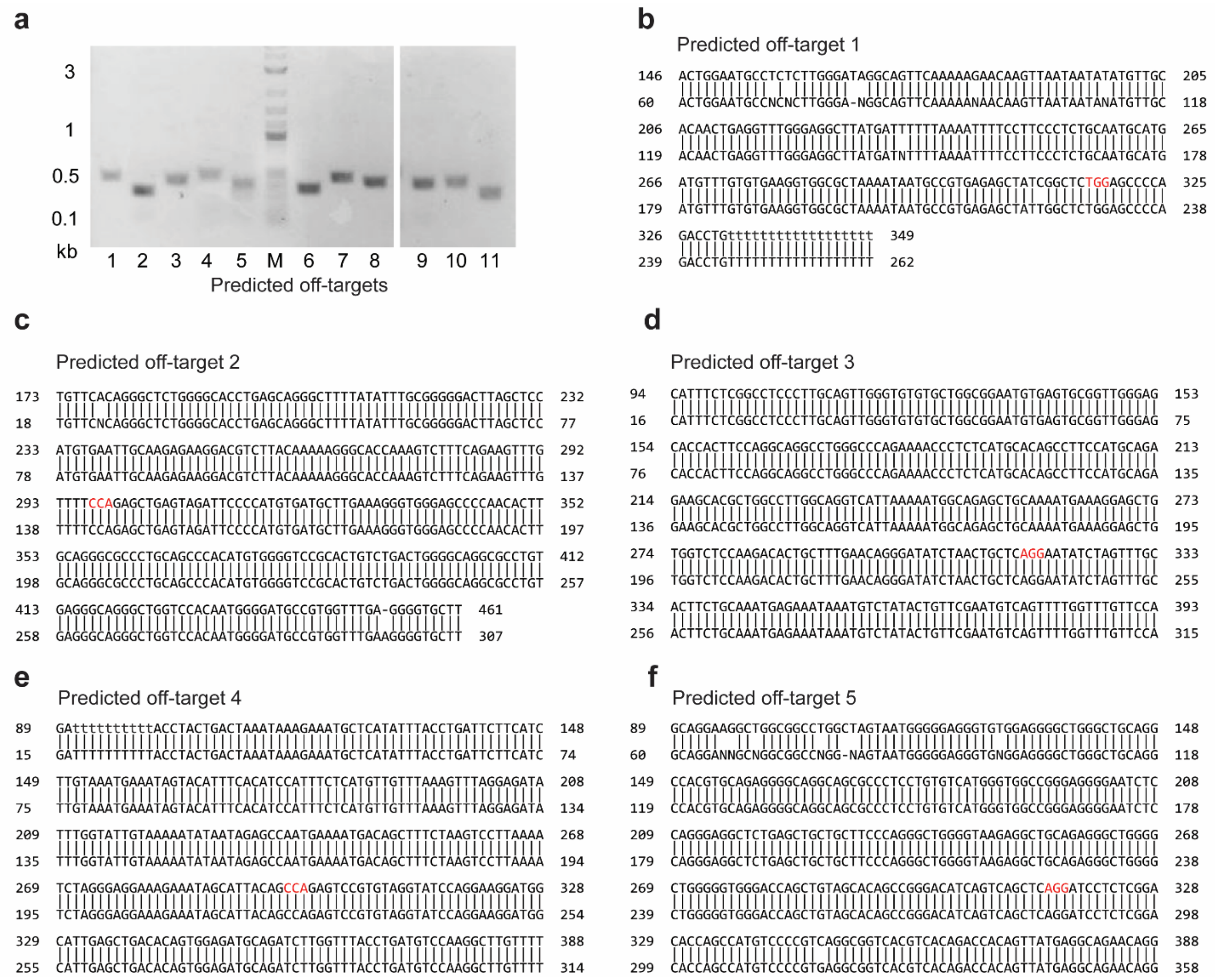
a
3
1
0.5
0.1
kb
1 2 3 4 5 M 6 7 8 9 10 11
Predicted off-targets
b
Predicted off-target 1
c
Predicted off-target 2
d
Predicted off-target 3
e
Predicted off-target 4
f
Predicted off-target 5

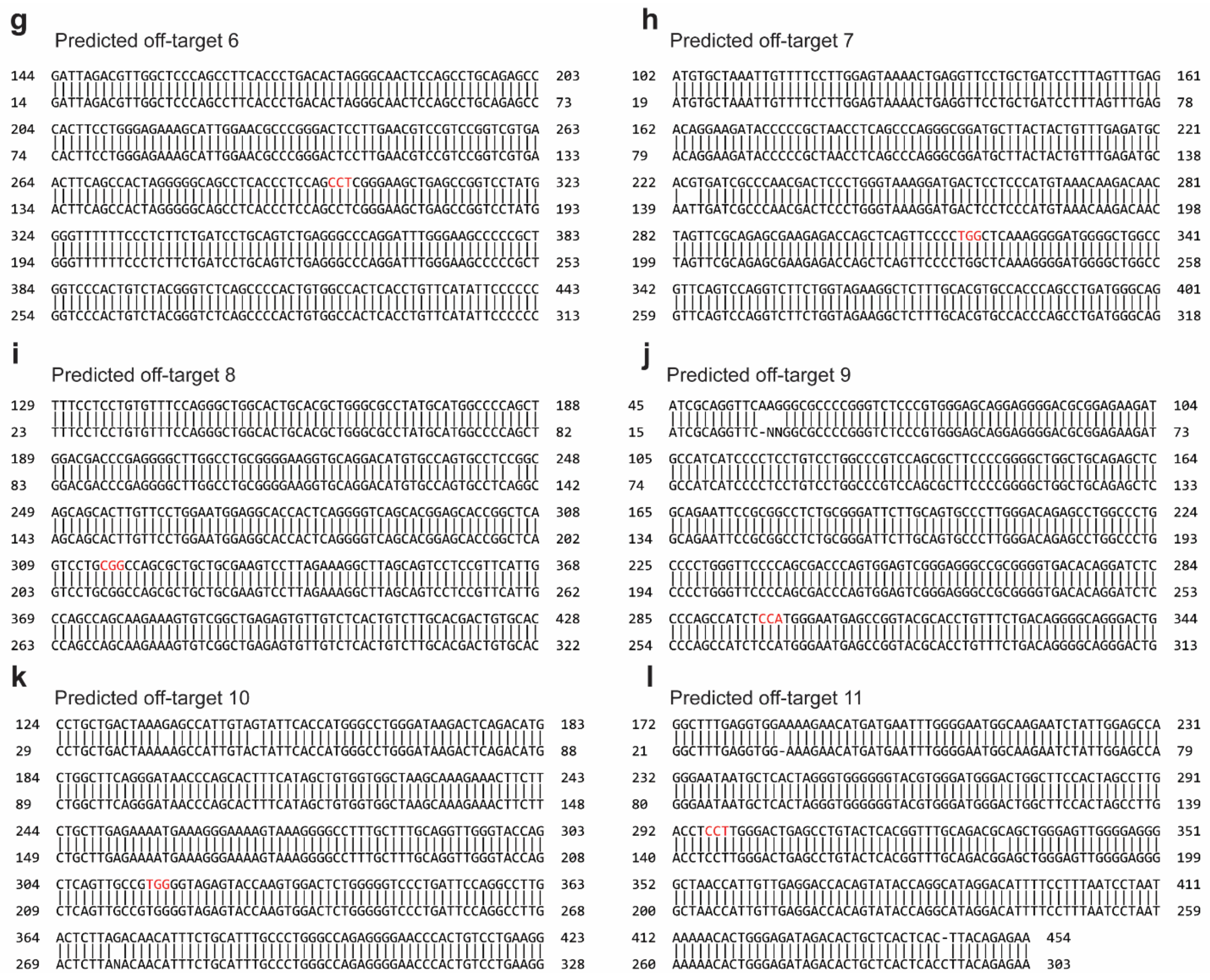

Supplementary Fig. 13 alt-RPL36 knock-out cells do not contain deletions or insertions at 11 predicted off-target sites. a Genomic PCR and sequencing of 11 off-target loci predicted by cas-offinder separated via agarose gel electrophoresis. **b-l** Sanger sequencing of 11 gel-purified genomic PCR products, followed with BLAST alignment. The NGG PAM sites of the predicted off-target guide RNA are indicated in red. Predicted off-targets 2, 4, 6, 9 and 11 target the antisense sequence. No mutations are observed near the NGG PAM sites.

qRT-PCR primers (5' - 3')			
Target genes	Use	Forward primer	Reverse primer
RPL36 v2	qRT-PCR	AGGTTGGAGGATGGTTGGTT	TGCTCACGTTCTTGGTCACTT
RPL36 v1 + v2	qRT-PCR	ACTGAAGGTCTCCAAGGAC	TCTTTATTTCAGGGAGAG
COL6A2	qRT-PCR	CTCCTCGGGACCAGGACTT	GGTGTCCAGCACGAAGTACA
EFNA3	qRT-PCR	ACAGCCCCATCAAGTTCTCG	GAGTGGGCGTGGAGATGTAG
PPP2R2C	qRT-PCR	CAGCTATGTGACTGAAGCTGAC	TAGTCAAACTCCGGCTCGTG
TMEM24	qRT-PCR	TTCCCAGTCTCTGTGACCCA	GATATGAGCAGCCCCTCACC
β-actin	qRT-PCR	AGGCACCAGGGCGTGAT	GCCCACATAGGAATCCTTCTGAC

Supplementary Table 1 qRT-PCR primers (5' - 3').

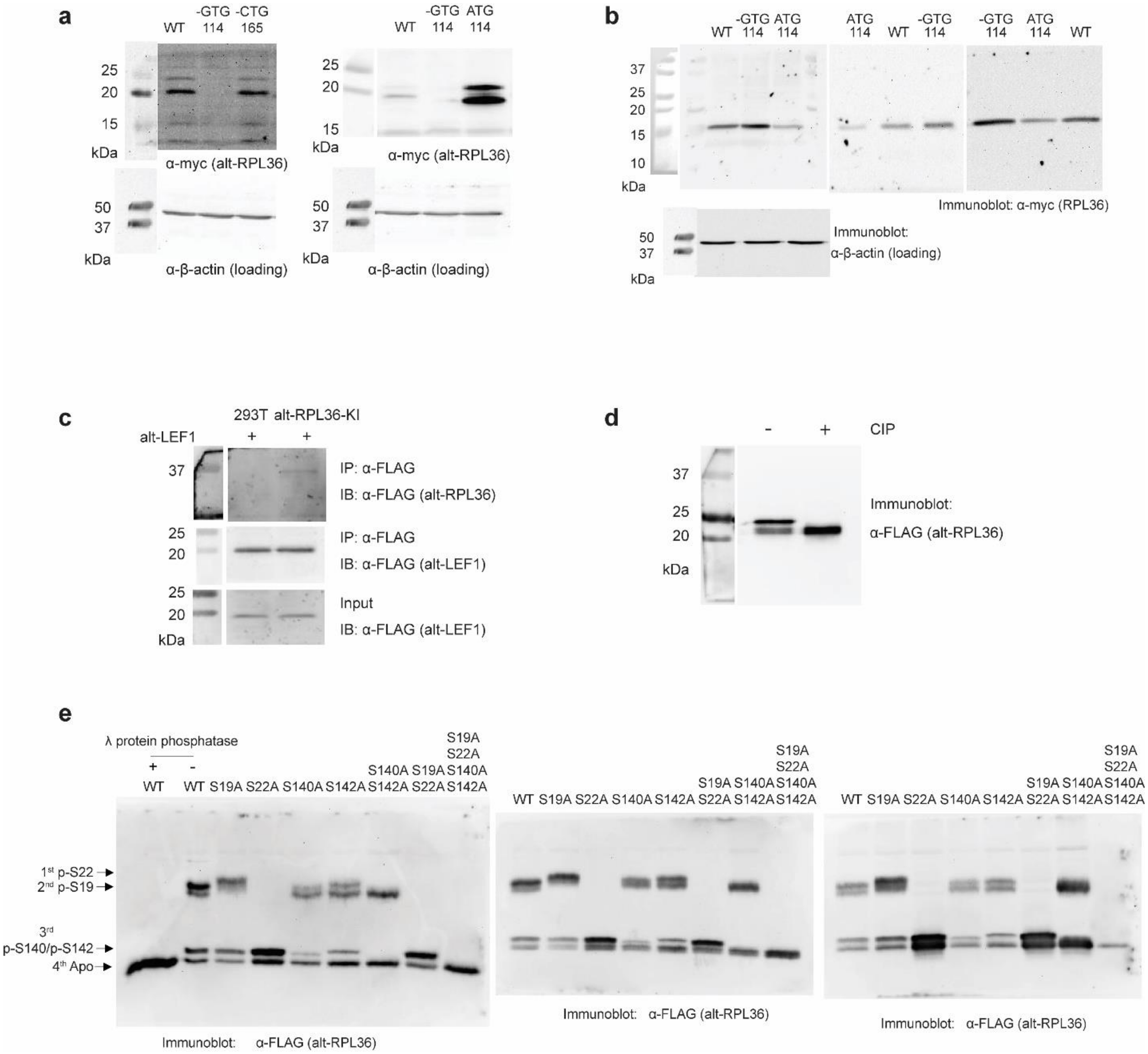

Supplementary Fig. 14 Uncropped blots from Figure 1, 2 and 3.

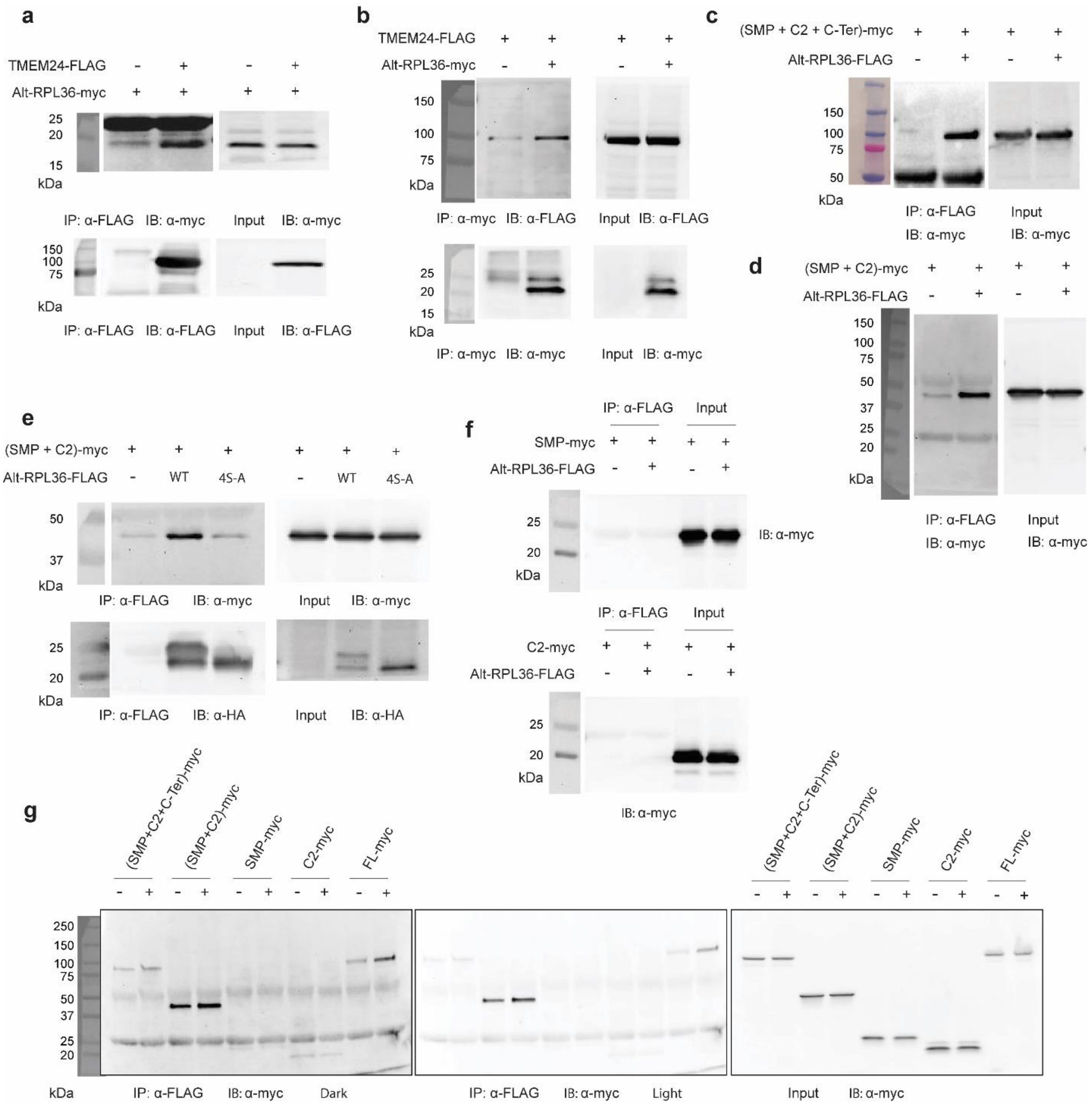

Supplementary Fig. 15 Uncropped blots from Figure 4 and Supplementary Figure 4.

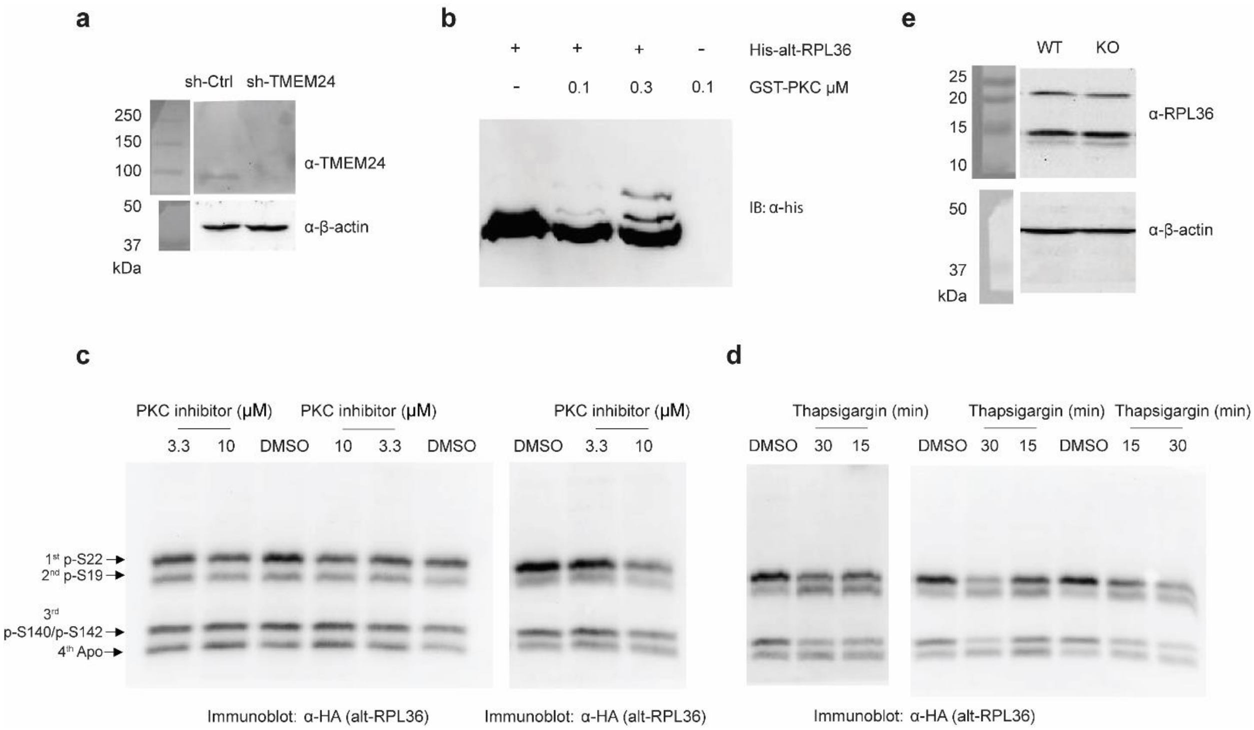

Supplementary Fig. 16 Uncropped blots from Supplementary Figures 5, 6 and 7.

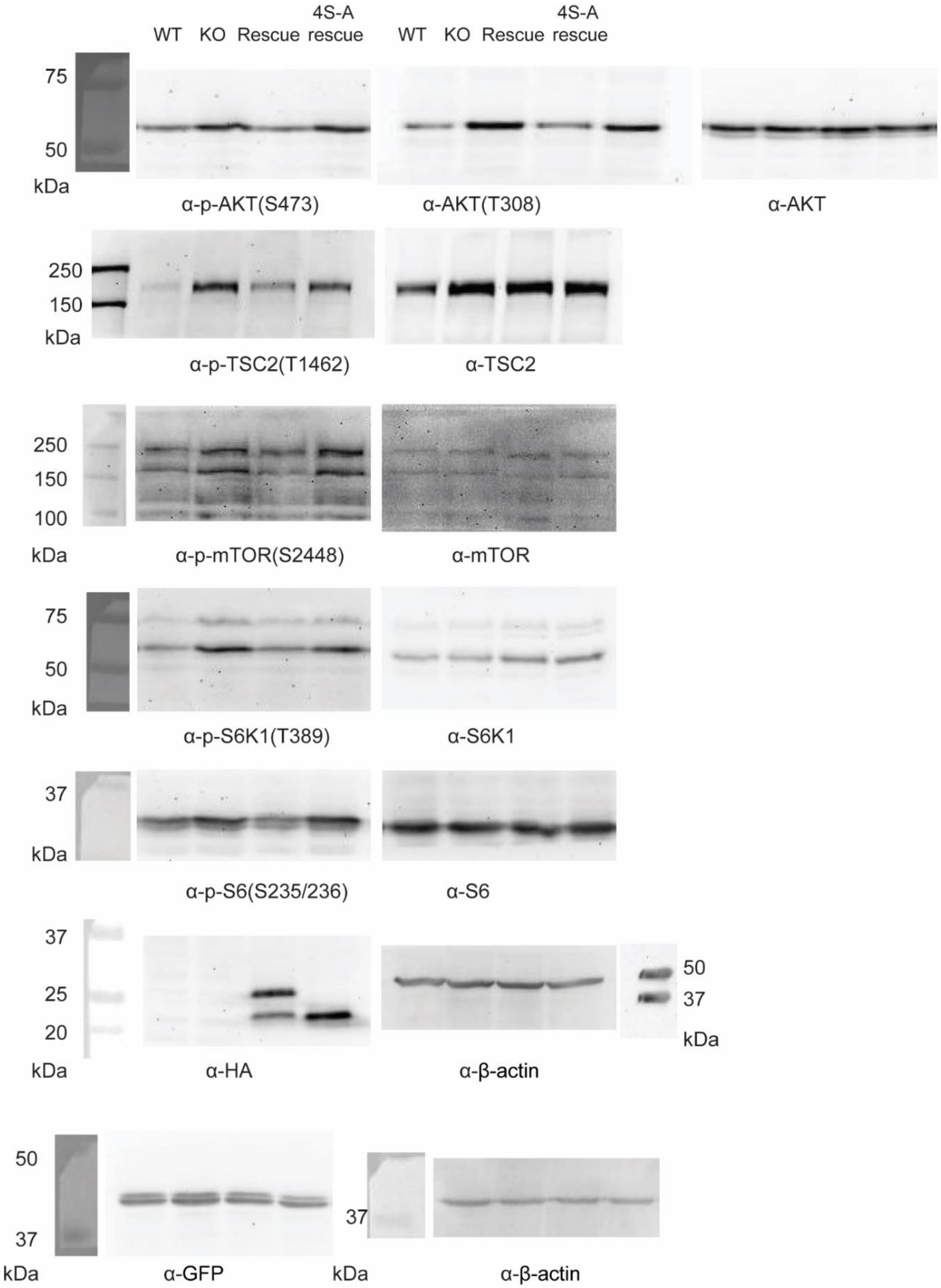

Supplementary Fig. 17 Uncropped blots from Figure 5.

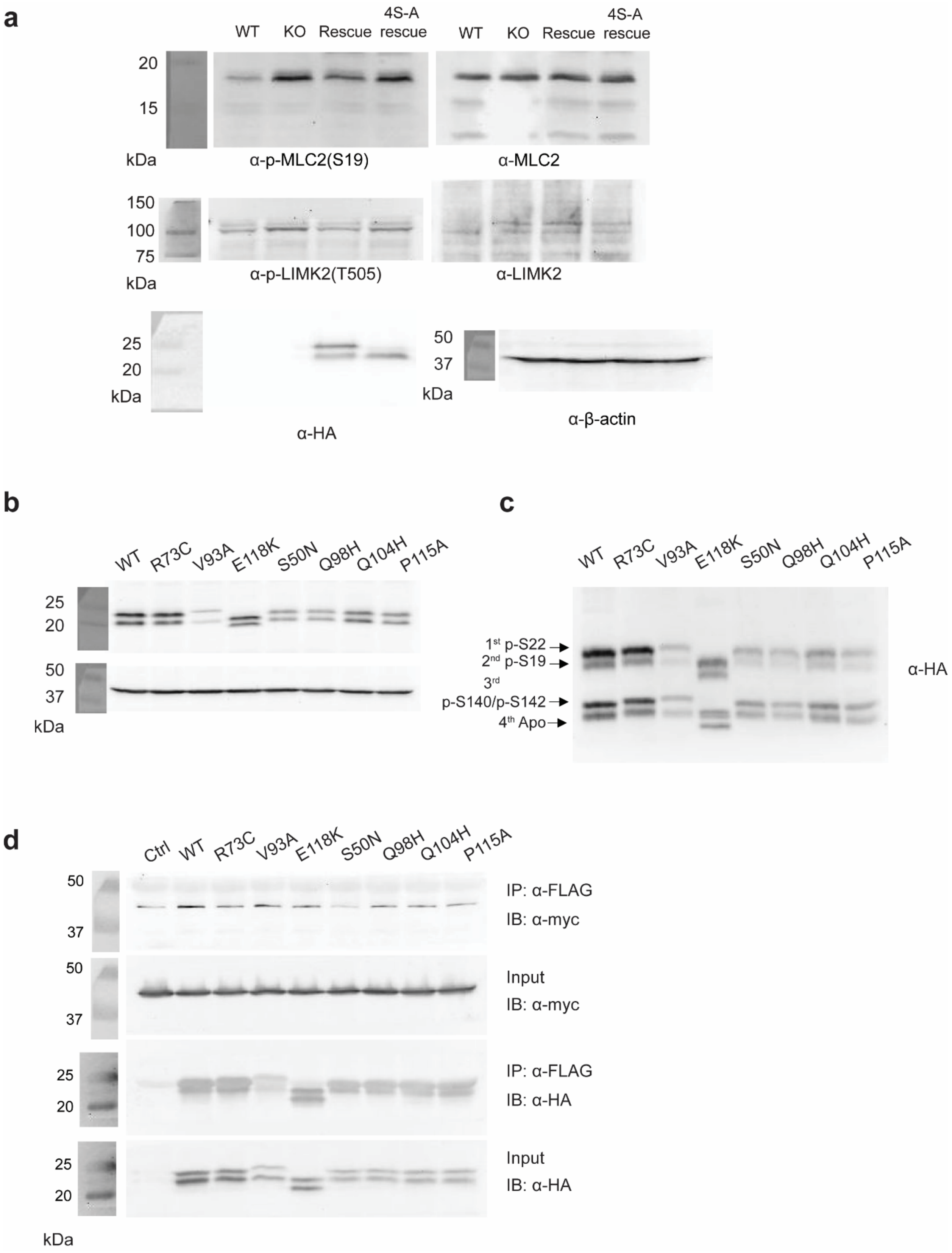

Supplementary Fig. 18 Uncropped blots from Figure 6 and Supplementary Figure 11.

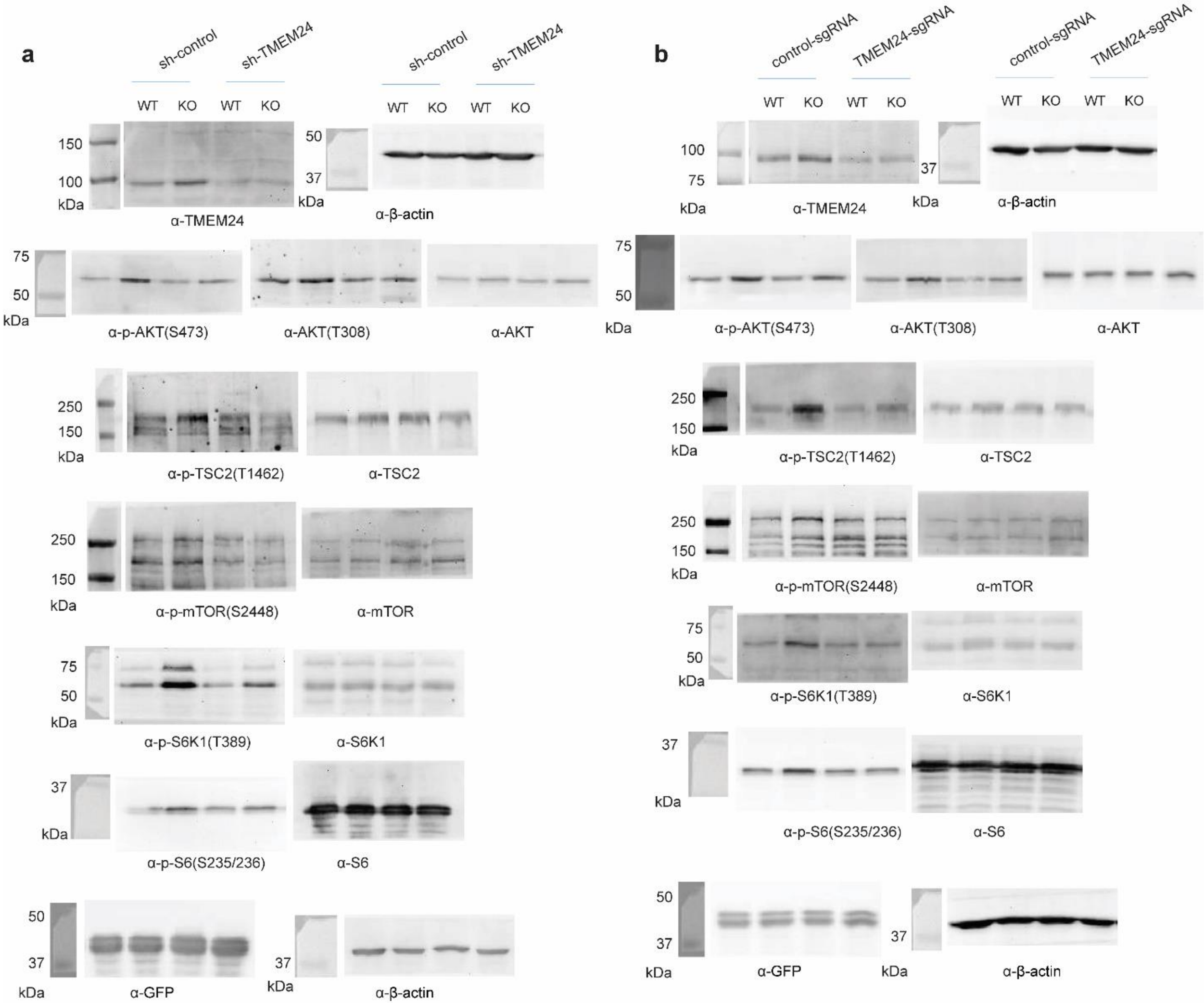

Supplementary Fig. 19 Uncropped blots from Figure 7 and Supplementary Figure 10.

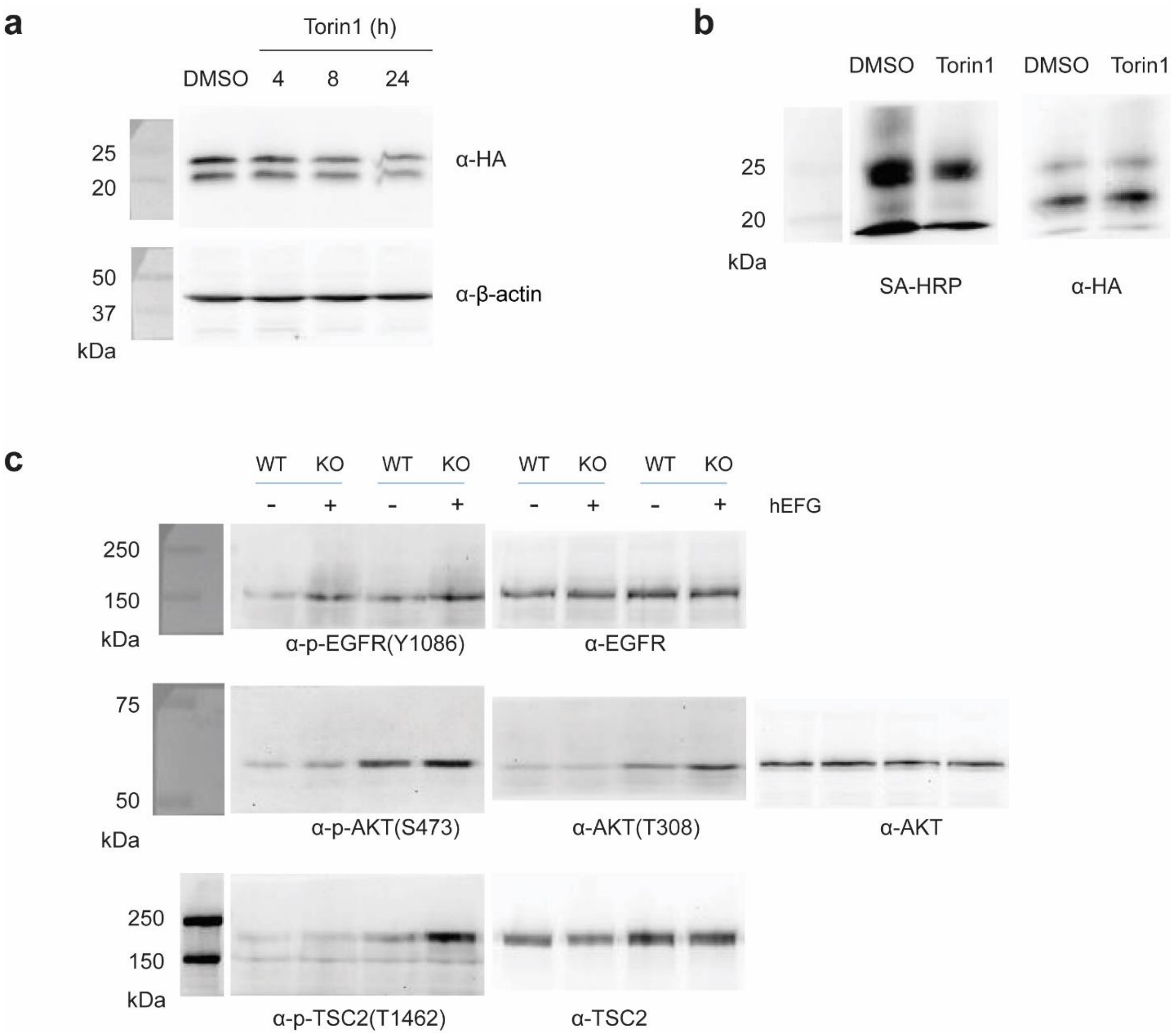

Supplementary Fig. 20 Uncropped blots from Supplementary Figure 8 and 9.
