# Supplementary material for: Alt-RPL36 downregulates the PI3K-AKT-mTOR signaling pathway by interacting with TMEM24
Source: Nat Commun. 2021 Jan 21;12:508. doi: 10.1038/s41467-020-20841-6 (PMC7820019; doi:10.1038/s41467-020-20841-6)
Supplement: Supplementary file 2 — Description of Additional Supplementary Files [file 41467_2020_20841_MOESM2_ESM.docx]

File Name: Supplementary Data 1

Description: Alt-RPL36 peptide- and protein-level evidence in HEK 293T, HT1080 and MOLT4 cells

File Name: Supplementary Data 2

Description: Phosphoproteomics: Alt-RPL36 phosphorylation peptide- and protein-level evidence

File Name: Supplementary Data 3

Description: Co-immunoprecipitation LC-MS/MS: protein-level evidence of alt-RPL36 interaction with TMEM24

File Name: Supplementary Data 4

Description: Structural variant analysis of alt-RPL36 KO and KI cell lines with Xdrop long-read DNA sequencing

File Name: Supplementary Data 5

Description: Genomic locations and primer sequences of eleven predicted off-target sites in alt-RPL36 KO
