# Supplementary material for: Alt-RPL36 downregulates the PI3K-AKT-mTOR signaling pathway by interacting with TMEM24
Source: Nat Commun. 2021 Jan 21;12:508. doi: 10.1038/s41467-020-20841-6 (PMC7820019; doi:10.1038/s41467-020-20841-6)
Supplement: Supplementary file 8 — Reporting Summary [file 41467_2020_20841_MOESM8_ESM.pdf]

nature research

Corresponding author(s): Sarah A. Slavoff

Last updated by author(s): December 11, 2020

Reporting Summary

Nature Research wishes to improve the reproducibility of the work that we publish. This form provides structure for consistency and transparency in reporting. For further information on Nature Research policies, see Authors & Referees and the Editorial Policy Checklist .

Statistics

For all statistical analyses, confirm that the following items are present in the figure legend, table legend, main text, or Methods section.

n/a	Confirmed	
☐	☒	The exact sample size (*n*) for each experimental group/condition, given as a discrete number and unit of measurement
☐	☒	A statement on whether measurements were taken from distinct samples or whether the same sample was measured repeatedly
☐	☒	The statistical test(s) used AND whether they are one- or two-sided<br>*Only common tests should be described solely by name; describe more complex techniques in the Methods section.*
☒	☐	A description of all covariates tested
☐	☒	A description of any assumptions or corrections, such as tests of normality and adjustment for multiple comparisons
☐	☒	A full description of the statistical parameters including central tendency (e.g. means) or other basic estimates (e.g. regression coefficient) AND variation (e.g. standard deviation) or associated estimates of uncertainty (e.g. confidence intervals)
☐	☒	For null hypothesis testing, the test statistic (e.g. *F*, *t*, *r*) with confidence intervals, effect sizes, degrees of freedom and *P* value noted<br>*Give P values as exact values whenever suitable.*
☒	☐	For Bayesian analysis, information on the choice of priors and Markov chain Monte Carlo settings
☒	☐	For hierarchical and complex designs, identification of the appropriate level for tests and full reporting of outcomes
☐	☒	Estimates of effect sizes (e.g. Cohen's *d*, Pearson's *r*), indicating how they were calculated

Our web collection on statistics for biologists contains articles on many of the points above.

Software and code

Policy information about availability of computer code

Data collection	Oxford Nanopore sequencing raw data (FAST5) was subjected to basecalling using Guppy v.3.4.5. Illumina's Pipeline software package was used for mRNA-seq base-calling for data collected via the HiSeq 2500 platform. Thermo Fisher Orbitrap Velos and Q Exactive Instrument Control Software was used for proteomic data collection.
Data analysis	RNA-seq data were mapped to the Human genome (hg19) using TopHat (v2.0.11). Differential expression were measured using Cufflinks (v.2.2.1). Mascot (version 2.5.1) and MaxQuant (version 1.5.8.3) were used for proteomics analysis. ImageJ was used for measuring mean intensity of PH-PLC-GFP. GraphPad Prism 7 and Excel 2013 were also used for data analysis.

For manuscripts utilizing custom algorithms or software that are central to the research but not yet described in published literature, software must be made available to editors/reviewers. We strongly encourage code deposition in a community repository (e.g. GitHub). See the Nature Research guidelines for submitting code & software for further information.

Data

Policy information about availability of data

All manuscripts must include a data availability statement. This statement should provide the following information, where applicable:

- Accession codes, unique identifiers, or web links for publicly available datasets
- A list of figures that have associated raw data
- A description of any restrictions on data availability

The mRNA-seq and Xdrop sequence data have been deposited in the NCBI Gene Expression Omnibus under accession GSE144979. Proteomics data were deposited under accession PXD018268 to the PRIDE repository. Proteomics data associated with Figures 1,3, and 4 are included as supplementary datas 1,3, and 4, respectively. Additional conservation data are available from the corresponding author on request.

Field-specific reporting

Please select the one below that is the best fit for your research. If you are not sure, read the appropriate sections before making your selection.

☒ Life sciences ☐ Behavioural & social sciences ☐ Ecological, evolutionary & environmental sciences

For a reference copy of the document with all sections, see nature.com/documents/nr-reporting-summary-flat.pdf

Life sciences study design

All studies must disclose on these points even when the disclosure is negative.

Sample size	Three biological replicates were performed unless noted otherwise. No statistical tests were used to determine sample size; the sample size was chosen based on previous reports on related experiments as sufficient to ensure reproducibility.
Data exclusions	Technical failures (imaging and Western blotting) were excluded based on pre-established criteria.
Replication	All attempts at replication were successful, and standard deviations were within expected ranges. All experiments were performed as three independent biological replicates, unless stated otherwise.
Randomization	Different cell passages were used for each biological replicates. Samples were randomly allocated into experimental groups.
Blinding	Blinding was not applicable for this study, as samples were processed identically through standard procedures.

Reporting for specific materials, systems and methods

We require information from authors about some types of materials, experimental systems and methods used in many studies. Here, indicate whether each material, system or method listed is relevant to your study. If you are not sure if a list item applies to your research, read the appropriate section before selecting a response.

Materials & experimental systems

n/a	Involved in the study	
☐	☒	Antibodies
☐	☒	Eukaryotic cell lines
☒	☐	Palaeontology
☒	☐	Animals and other organisms
☒	☐	Human research participants
☒	☐	Clinical data

Methods

n/a	Involved in the study	
☒	☐	ChIP-seq
☒	☐	Flow cytometry
☒	☐	MRI-based neuroimaging

Antibodies

Antibodies used	Primary antibodies for Western blotting include the following anti-FLAG (Sigma, F3165); anti-myc (Rockland, 600-401-381, Cell Signaling, 2276 (mouse), or Cell Signaling, 2278 (rabbit)); anti-HA (Invitrogen, 71-5500); anti-His (Thermo Fisher, MA1-21315); anti-V5 (Cell Signaling, 13202); anti-β-actin (Invitrogen, BA3R); anti-p-mTOR(S2448) (Cell Signaling Technology, 2971); anti-mTOR (Cell Signaling Technology, 2972); anti-p-AKT(S473) (Cell Signaling Technology, 9271); anti-p-AKT(T308) (Cell Signaling Technology, 4056); anti-AKT (Cell Signaling Technology, 9272); anti-p-TSC2(T1462) (Cell Signaling Technology, 3617); anti-TSC2 (Cell Signaling Technology, 4308); anti-p-S6K1(T389) (Cell Signaling Technology, 9234); anti-S6K1 (Cell Signaling Technology, 9202); anti-p-S6(S235/236) (Cell Signaling Technology, 2211); anti-S6 (Cell Signaling Technology, 2217); anti-p-MLC2(S19) (Cell Signaling Technology, 3671); anti-MLC2 (Cell Signaling Technology, 3672); anti-p-LIMK2(T505) (Cell Signaling Technology, 3841); anti-LIMK2 (Cell Signaling Technology, 3845); anti-p-EGFR(Y1068) (Abcam, ab40815); anti-EGFR (Abcam, ab52894); anti-RPL36 (Bethyl Laboratories, A305065A-M); anti-TMEM24 (a gift from Pietro De Camilli, Yale). Immunoprecipitation was performed with the following antibody beads: anti-FLAG M2 affinity gel (Sigma, A2220); anti-myc tag agarose beads (Sigma, A7470); anti-HA tag magnetic beads (Thermo Fisher, 88836).
Validation	Validation was performed by respective suppliers. All primary antibodies used for Western blotting were validated for Western blotting application in human cells. All antibody beads were validated for immunoprecipitation in human cells.

Eukaryotic cell lines

Policy information about cell lines

Cell line source(s)	Wild-type HEK 293T (ATCC), alt-RPL36 knockout (KO), "rescue" cell line in which the alt-RPL36 coding sequence was stably reintroduced on the KO background, as well as "4S-A rescue" cells, in which the non-phosphorylatable mutant of alt-RPL36 was reintroduced on the KO background.

Authentication	HEK 293T cells were authenticated by ATCC through STR profiling , knockout cell line was validated by DNA sequencing of PCR products amplified from genomic locus and by RNA-seq, and Rescue cell lines were validated by Western blotting.
Mycoplasma contamination	All cell lines were tested negative for mycoplasma using the ATCC universal mycoplasma detection kit.
Commonly misidentified lines (See ICLAC register)	No commonly misidentified cell lines were used.
